# Mesenchymal Stem Cell-Derived Extracellular Vesicles: Opportunities and Challenges for Clinical Translation

**DOI:** 10.3389/fbioe.2020.00997

**Published:** 2020-09-10

**Authors:** Marie Maumus, Pauline Rozier, Jérémy Boulestreau, Christian Jorgensen, Danièle Noël

**Affiliations:** ^1^IRMB, University of Montpellier, INSERM, CHU Montpellier, Montpellier, France; ^2^Bauerfeind France, IRMB, Montpellier, France; ^3^Clinical Immunology and Osteoarticular Diseases Therapeutic Unit, Department of Rheumatology, Lapeyronie University Hospital, Montpellier, France

**Keywords:** mesenchymal stem cells, extracellular vesicles, regenerative medicine, therapy, clinical translation

## Abstract

Extracellular vesicles (EVs), including exosomes and microvesicles, derived from mesenchymal stem/stromal cells (MSCs) exert similar effects as their parental cells, and are of interest for various therapeutic applications. EVs can act through uptake by the target cells followed by release of their cargo inside the cytoplasm, or through interaction of membrane-bound ligands with receptors expressed on target cells to stimulate downstream intracellular pathways. EV-based therapeutics may be directly used as substitutes of intact cells or after modification for targeted drug delivery. However, for the development of EV-based therapeutics, several production, isolation, and characterization requirements have to be met and the quality of the final product has to be tested before its clinical implementation. In this review, we discuss the challenges associated with the development of EV-based therapeutics and the regulatory specifications for their successful clinical translation.

## Introduction

Extracellular vesicles (EVs), including exosomes and microvesicles, are nanoscale vesicles that are released by all cell types and act as signaling/communication agents between adjacent or distant cells. The transmission of information to a multitude of cells and locations confers them important roles in both physiological and pathological processes. EVs derived from mesenchymal stromal/stem cells (MSCs) display similar functions as their parental cells and show therapeutic efficacy in many non-clinical models (Keshtkar et al., 2018). MSC-derived EVs (MSC-EVs) exert their functions through the transfer of their cargo (i.e., proteins, lipids, and nucleic acids such as mRNA, micro-RNAs, long non-coding RNAs, DNA, and metabolites) (Busatto et al., 2019; Qiu et al., 2019). They can be used as therapeutic tools either in naïve form, as substitutes of intact cells, or after modification for targeted drug delivery. However, for clinical applications, EV safe and effective production systems and rigorous quality control are needed before the release of clinical batches. The *International Society for Extracellular Vesicles* (ISEV) and the *European Network on Microvesicles and Exosomes in Health and Diseases* (ME-HaD) have highlighted a number of safety and regulatory requirements that must be considered for the clinical applications of EV-based therapeutics (Lener et al., 2015). In the present review, we summarize the recent developments in EV production for pharmaceutical manufacturing, and discuss the regulatory issues associated with their clinical application.

## Definition and Characterization of Extracellular Vesicles

Extracellular vesicles are defined as particles that are delimited by a lipid bilayer, cannot replicate, and are released from the cell (Thery et al., 2018). At least three EV types can be characterized on the basis of their biogenesis pathway: (i) exosomes (small particles of endocytic origin with a diameter of 30–150 nm), (ii) microvesicles or microparticles (generated from the plasma membrane by direct budding; diameter of 150–500 nm), and (iii) apoptotic bodies (vesicles of 800–5000 nm in diameter, formed via membrane blebbing of apoptotic cells) (He et al., 2018). However, due to the overlapping sizes among EV subtypes and the frequent use of isolation techniques that rely on size-based separation, the ISEV recently recommended to define them as small vesicles (EVs < 200 nm), and medium or large EVs (EVs > 200 nm) (Thery et al., 2018). This definition is not comprehensive, particularly concerning EV biogenesis, but probably it represents the best option for classifying EVs that are mainly isolated according to their size. A more accurate definition will require the development of devices that allow EV isolation with high yields based on the presence of specific biomarkers and that are compatible with large-scale production.

Extracellular vesicles are secreted by all human and non-human cell types and can be divided into plant-derived EVs, bacterial/fungal/parasitic EVs, and animal product-derived EVs (Schuh et al., 2019). They are key components of the local environment through intercellular communication pathways, and also of the systemic environment through their release into the fluids of complex organisms. In animals and humans, they can be isolated from all body fluid types: blood, urine, breast milk, synovial liquid, amniotic fluid, cerebrospinal fluid, saliva,… (Schuh et al., 2019). EVs convey large numbers of molecules (e.g., proteins, mRNA, non-coding RNAs, and lipids) that mediate different functions, depending on the cells from which they originate. They transfer signals to recipient cells through different mechanisms: receptor-ligand interactions, direct membrane fusion, and endocytosis/phagocytosis (van Niel et al., 2018). They act in a paracrine and endocrine manner, and can be also taken up by their cells of origin. Therefore, EVs have important roles in both physiological and pathological processes.

## Potential Therapeutic Use of Mesenchymal Stem Cell-Derived Extracellular Vesicles

Extracellular vesicles from various cell sources have different physiological functions and therefore, may have different therapeutic applications. They were first investigated as vaccines to enhance the antitumor response using antigen-presenting cells, primarily dendritic cells loaded with tumor antigens, and then as vaccines for infectious and allergic diseases (for review, see Markov et al., 2019; Zurita et al., 2019). Subsequently, the detection of EVs with increased concentrations and differential cargoes in body fluids from patients with different pathological conditions led to much research on the potential use of EV proteins and RNA molecules as biomarkers of different diseases (Lasser, 2015). Finally, due to their capacity to carry large numbers of active molecules, EVs can be exploited as drug delivery systems, and can be chemically or biologically engineered to deliver enhanced or broaden therapeutic agents. Indeed, EVs act as “logistics shuttles” that show high stability in the bloodstream, specific targeting capacities (like their parent cells), and capacity to pass through physiological barriers (the blood brain barrier, for example). Indeed, MSC-derived EVs were shown to strongly inhibit lymphocyte proliferation and antibody production by targeting B-cells in heart failure (van den Hoogen et al., 2019). Chemical modification of EVs by addition of RGD peptides conjugated onto EV surfaces led the EVs to pass the blood brain barrier and target brain cells after ischemic stroke (Tian et al., 2018). EVs can also be loaded with drugs by cell transfection and genetic expression of a candidate gene, or by drug encapsulation after their isolation [(Mao et al., 2019; He et al., 2020; Ou et al., 2020); for review, see Crivelli et al. (2017)]. MSC-EVs were extensively characterized as drug delivery platform and shown to have greater internalization capabilities than commercial liposomes (Le Saux et al., 2020). The interest of using MSC-EVs loaded with doxorubicin by electroporation to target murine breast cancer cells or osteosarcoma cells has been demonstrated (Gomari et al., 2019; Wei et al., 2019). Interestingly, MSCs can entrap drug-loaded nanoparticles and release EVs that contain the nanoparticles enabling to combine MSC-based regenerative therapy to pharmaceutical nanomedicine (Perteghella et al., 2017).

The interest of using MSC-EVs for clinical applications is related to the variety of molecules with therapeutic functions they can carry and the fact that their cargo is naturally protected from degradation in the circulation (Tsui et al., 2002). EVs isolated from autologous MSCs are non-immunogenic. Although they should be poorly immunogenic in the case of allogenic injection thanks to the immunomodulatory molecules they convey, it is still unclear whether EVs contain major histocompatibility complex (MHC) molecules that might elicit alloimmune responses (Crivelli et al., 2017; Lohan et al., 2017). Numerous reports have highlighted the functional properties of MSCs and MSC-EVs using *in vitro* assays, and identified many factors involved in their functions (for review, see Doorn et al., 2012; Maumus et al., 2013; Clark et al., 2014; Glenn and Whartenby, 2014; Burrello et al., 2016; Abbasi-Malati et al., 2018). The possibility of using MSC-EVs to eliminate or reduce the clinical symptoms of several diseases has been widely assessed in animal models. A recent review of the literature discussed the applications of EVs from umbilical cord-derived MSCs (UC-MSCs) in various diseases (Yaghoubi et al., 2019). EVs isolated from different MSC sources have shown efficacy in non-clinical models of neurological diseases, particularly epilepsy (Xin et al., 2013; Long et al., 2017), post-traumatic brain injury (Zhang et al., 2015), brain damage in pre-term neonates (Ophelders et al., 2016; Drommelschmidt et al., 2017; Sisa et al., 2019), and stroke (Doeppner et al., 2015). In animal models, MSC-EVs have been used to treat myocardial infarction (Lai et al., 2010; Bian et al., 2014) and for ischemic injury prevention in chronic renal failure (Gregorini et al., 2017). MSC-EVs are also efficient for the management of acute conditions, such as acute renal failure (Bruno et al., 2009) and respiratory failure (Zhu et al., 2014; Monsel et al., 2015; Monsel et al., 2016). MSC-EVs can reduce clinical symptoms in murine models of osteoarthritis and rheumatoid arthritis (Cosenza et al., 2017, 2018). Finally, MSC-EVs are effective in liver regeneration, as well as in experimental infectious conditions and ophthalmic diseases (Li et al., 2013; Tan et al., 2014; Tan et al., 2016; Bai et al., 2017; Chen et al., 2017; Chang et al., 2018). In conclusion, many studies have demonstrated the therapeutic efficacy of MSC-EVs in animal models and their potential is now evaluated in human clinical trials.

As it is generally accepted that MSCs from different tissue sources and from different donors display qualitatively different functional capacities, EVs isolated from different MSCs also should present differences in their cargo and related properties (Baglio et al., 2015). However, only few studies compared EVs from MSCs isolated from different sources. It has been recently reported that EVs from adipose tissue-derived MSCs (AD-MSCs) and from cardiac MSCs exhibit more potent angiogenic capacities than bone marrow-derived MSCs (BM-MSCs) (Chance et al., 2020; Kang et al., 2020). In addition, the capacity of EV production and secretory profile are higher in BM-MSCs than AD-MSCs, supporting a differential activity of EVs from different MSCs (Villatoro et al., 2019). Similarly, the expression of surface markers and function vary in MSCs from different species. Indeed, although MSC immunosuppressive capacity may cross the species barriers, different mechanisms of action are reported: through soluble factors for human MSCs and through cell–cell contacts for rodent MSCs (Uder et al., 2018). To our knowledge, no study has compared EVs from different species yet. Nevertheless, it is obvious that EVs from different species are different. This implies that the conclusions of pre-clinical studies that use human EVs in animal models have to be taken with cautions.

Another important notion for EV therapeutic applications is the definition of the minimal dose for effective clinical outcome in patients. Like parental MSCs, the effective dose of EVs is dependent on the biological activity, which can be defined on the basis of the number of particles or the quantity of bioactive proteins or RNAs. However, the protein or RNA content may quantitatively and qualitatively vary in function of MSC culture conditions or activation status and EV production method. In general, a dose-dependent effect of MSC-EVs has been observed using different functional assays (Cosenza et al., 2018; Bari et al., 2019a; Dal Collo et al., 2020). For instance, 50 μg of MSC-EVs are needed to induce the proliferation and differentiation of neural stem cells to oligodendrocytes (Otero-Ortega et al., 2020), while 10 μg of placenta-derived MSC-EVs are sufficient to increase the migration and tube formation of placental microvascular endothelial cells (Salomon et al., 2013). These differences in the dose needed for bioactivity can be related to the MSC source, the method used for EV isolation, or the mechanism of action (MoA) of EVs. The definition of the MoA for a specific therapeutic indication should allow designing a reproducible and reliable functional *in vitro* assay to determine the EV protein or RNA effective concentration, as proposed in Dal Collo et al. (2020). However, *in vitro* potency assays do not necessarily predict the therapeutic effect *in vivo*, and even less the patients’ outcome in clinical trials.

The minimal effective dose of EVs could be determined using a relevant pre-clinical model (ideally a large animal model) for a specific therapeutic application. The therapeutic dose of EVs is usually in the range of 10–100 μg of proteins in mouse models (Riau et al., 2019). For example, a dose of 50 μg of EVs was sufficient to enhance protection and brain repair in a rat model of subcortical ischemic stroke, compared with 100 and 200 μg of EVs (Otero-Ortega et al., 2020). This dose was also the smallest effective dose identified in a functional *in vitro* assay. However, the most efficient dose is not always the highest dose, as shown for MSCs in a model of systemic sclerosis (Maria et al., 2016). Interestingly, it was reported that EVs isolated from non-pigmented ciliary epithelium display enhanced pro-MMP9 activities at high doses, but significantly reduce β-catenin expression and GSK-3 phosphorylation only at low doses (Tabak et al., 2018). This concentration-dependent effect of EVs might be related to different interaction modes with the target cells (e.g., direct binding to cell membrane receptors or internalization).

Moreover, investigating different administration routes may help to reduce the effective dose, if the accessibility of the target tissue is increased. Indeed, as the route of administration determines EV biodistribution, increasing the uptake of exogenous EVs by a targeted organ can enhance their efficacy (Di Rocco et al., 2016). Unlike intravenous injection, EV administration by the intraperitoneal or subcutaneous route results in higher accumulation in pancreas and gastrointestinal tract and in lower concentrations in liver and spleen (Wiklander et al., 2015). In addition, EV uptake is potentiated by the concomitant presence of extracellular proteins, for instance albumin (Schneider et al., 2017). Moreover, EV dose also can affect their biodistribution, as indicated by the inverse correlation between intravenous injection of increasing EV concentrations and their accumulation in liver (Wiklander et al., 2015).

In conclusion, the minimal effective doses of EVs can be determined by *in vivo* studies and these findings can be extrapolated for human use. EV dose and also the route, timing, and frequency of administration need to be carefully investigated for optimal and safe EV delivery in patients, as discussed elsewhere (Bari et al., 2019b).

## Clinical Applications of Mesenchymal Stem Cell-Derived Extracellular Vesicles

A total of nine clinical trials can be identified in the ClinicalTrials.gov database when using the keywords “exosomes” and/or “extracellular vesicles” and focusing on MSC-EVs (Table 1). Six of them are still recruiting or are completed, two trials are not recruiting yet, and one has an unknown status.

**TABLE 1:** Clinical trials evaluating MSC-EV therapies.

Disease	EV type	Administration route	Injected dose and time of injection	Cell source	Trial phase	Control group	Status	Number of patients	Ref/NTC
Type 1 diabetes	Exo and MV	IV	Eq of SN from 1.2-1.5 × 10^6^ cells/kg body weight (day 0)	UC-MSCs	1	No	Unknown	20	NCT02138331
Macular holes	Exo	Local	20 μg or 50 μg Eq proteins (day 0)	UC-MSCs	1	Yes	Recruiting	44	NCT03437759
Bronchopulmo-nary dysplasia	Not indicated	IV	20, 60, or 200 pmol/kg (day 0)	BM-MSCs	1	Yes	Recruiting	18	NCT03857841
Dystrophic epidermolysis bullosa	Exo	Local	Not indicated (once a day for 60 days)	Not indicated	1/2	Yes	Not yet recruiting	30	NCT04173650
Dry eye (graft versus host disease)	Exo	Local	10 μg Eq proteins/drop, 4 times a day, 14 days	UC-MSCs	1	No	Recruiting	27	NCT04213248
Ischemic stroke	Exo	Local	200 μg Eq proteins (day 0)	miR-124 over expressing MSCs	1/2	No	Completed	5	NCT03384433
Pancreatic cancer	Exo	IV	Unspecified	MSCs loaded with KrasG12D siRNA	1	No	Not yet recruiting	28	NCT03608631
Graft versus host disease	Small size EVs	IV	4 units (1 unit = Eq of SN from 4 × 10^7^ cells) (day 0)	BM-MSCs	NA	NA	Completed	1	Kordelas et al., 2014
Chronic kidney disease	Total EVs	IV (1st)/IA (2nd)	100 μg Eq proteins/kg (x2) (day 0)	UC-MSCs	2/3	Yes	Completed	40	Nassar et al., 2016

**EVs, extracellular vesicles; Eq, equivalent dose; Exo, exosomes; IV, intravenous; IA, intra-arterial; MV, microvesicles; SN, supernatant; BM-MSC, bone marrow-derived mesenchymal stem cells; UC-MSC, umbilical cord-derived mesenchymal stem cells; NA, not applicable*.*

The first phase I clinical trial was initiated in 2014 with the aim of evaluating the safety of EVs isolated from UC-MSCs in 20 patients with type 1 diabetes. Patients received a systemic injection of exosomes at day 0 and of microvesicles at day 7, and the effect on the total daily requirement of insulin was evaluated at 3 months. The status of the trial is unknown. In 2017, another phase I study enrolled patients with large and refractory macular holes (MH). This randomized and controlled study has included 44 patients who received 20 or 50 μg of UC-MSC-derived exosomes in the vitreous cavity close to the MH, after pars plana vitrectomy and internal limiting membrane peeling. This study is still recruiting. The treatment efficacy is evaluated by assessing MH closure by optical coherence tomography, at 24 weeks post-treatment. More recently, a safety and tolerability study was performed in pre-term neonates (born before gestational week 27) at high risk of bronchopulmonary dysplasia (BPD). This multi-center controlled double-bind trial included 3 to 14-day-old neonates (*n* = 18) who received 20, 60, or 200 pmol phospholipid of BM-MSC-EVs (UNEX-42)/kg body weight by intravenous injection. Safety was the primary endpoint, but BPD incidence and severity were also determined (secondary endpoints). A phase 1/2 multi-center randomized study to evaluate the effectiveness and safety of daily local injections of MSC-EVs (AGLE-103) in 30 patients with dystrophic epidermolysis bullosa was registered in November 2019. The primary endpoint is the safety and efficacy of wound closure at 8 months after treatment. The last phase 1/2 clinical study recorded in December 2019 will assess the alleviation of dry eye symptoms in 27 patients with chronic graft versus host disease (GVHD) after local treatment with UC-MSC-derived exosomes four times per day for 14 days. The treatment safety and efficacy will be evaluated at different time points by measuring the changes in the ocular surface disease index.

Two phase I clinical trials evaluate genetically engineered MSC-EVs. The first trial assessed EVs isolated from miR-124-overexpressing MSCs in five patients with acute ischemic stroke. Patients received 200 μg of total EV protein by stereotaxis, 1 month after the stroke. The incidence of adverse events was the primary outcome measure, but efficacy was also assessed using a Modified Rankin Scale after 12 weeks of treatment. The trial has recently been completed, but results are not available yet. The second clinical trial will evaluate MSC-derived exosomes loaded with small interfering RNAs against KRAS G12D (iExosomes) in patients with metastatic pancreatic cancer. Patients (*n* = 28) will receive the treatment by intravenous route at days 1, 4, and 10, and then every 14 days for up to three courses in the absence of adverse events or unfavorable disease outcome. The study aim is to identify the maximum tolerated dose and the dose-limiting toxicities of iExosomes, but has not recruited patients yet.

There are only two publications on the use of MSC-EVs in the clinic. The first article reported the case of one patient with therapy-refractory GvHD who received four units of BM-MSC-derived small EVs by intravenous injections (Kordelas et al., 2014). One unit of EVs was defined as the EV fraction recovered from the supernatant of 4 × 10^7^ BM-MSCs conditioned for 48 h and isolated by filtration using 0.22 μm filter membranes, precipitation with polyethylene glycol, and a final ultracentrifugation at 100.000 g for 2 h. To reduce the potential side effects, the patient initially received one tenth of a unit, and then progressively increasing unit amounts every 2–3 days to a total of 4 units. No adverse event was observed and clinical symptoms were remarkably improved within 14 days after EV administration, suggesting the safety and potential efficacy of this EV-based treatment. The second article concerned a randomized placebo-controlled clinical trial that evaluated the safety and efficacy of UC-MSC-EVs in 40 patients with stage III and IV chronic kidney disease (Nassar et al., 2016). EVs were collected from UC-MSC conditioned supernatant using two ultracentrifugation steps at 100,000 g for 1 h. Patients received two injections of 100 μg EVs/kg body weight 1 week apart, the first one by the intravenous route and the second one through the intra-renal arteries. No adverse event was recorded. The overall renal function significantly improved during the 12-month follow-up period. Interestingly, TGFβ1 and IL10 levels significantly increased concomitantly with the clinical improvement, suggesting immune modulatory regulation. Although few clinical results are available, pre-clinical data and early clinical results on EV-based therapeutics are very encouraging. However, it is important to stress that these clinical trials are mostly phase 1 studies on the feasibility and safety of EV administration for different clinical applications. Only one phase 2/3 trial has been completed and showed the safety (primary endpoint) and efficacy (secondary endpoint) of UC-MSC-EVs in patients with chronic kidney disease, as indicated by the reduction of serum creatinine level by 50% and the twofold increase in eGFR (Nassar et al., 2016). Randomized, double-blinded phase 2 and 3 clinical trials are required to definitively demonstrate MSC-EV efficacy and therapeutic interest.

## Challenges for the Industrial Production of GMP-Grade Extracellular Vesicles

The main challenge linked to the industrialization of EV-based therapeutics for regenerative medicine is to define new manufacturing strategies under Good Manufacturing Practice (GMP) for EV scalable production and isolation. Standardized operating procedures (SOPs) using reproducible and standardized assays are mandatory to manufacture a defined and qualified EV product because each manufacturing procedure will generate a different product. To reach this goal, major questions have to be addressed early in the product development: (i) how to manufacture EVs; (ii) how to characterize and qualify the final product; and (iii) how to organize the product storage in order to maintain its stability.

### Manufacturing

#### MSC Sources

Several tissue sources of MSCs can be used, such as BM, adipose tissue, synovial membrane, UC. These MSCs have been tested in various *in vitro* functional assays and in a large number of non-clinical disease models where MSC-EVs have shown therapeutic efficacy [for review see D’Arrigo et al. (2019)]. Nevertheless, no comparative study identified the most efficient MSC source for EV production, in terms of quantities or functional activities. Primarily two sources of MSCs (UC- and BM-MSCs) have been tested in clinical trials, but for different applications (Table 1). Therefore, the first question for EV manufacturing is the identification of the best MSC source(s) for a specific clinical application, and more data are necessary to answer this question. The best MSC source can be determined by identifying the most relevant MoA for the targeted therapeutic activity. For example, if an anti-inflammatory or pro-angiogenic function is envisioned, AD-MSCs or UC-MSCs might be preferred to BM-MSCs. It is nevertheless recommended to determine the best MSC source experimentally by comparing MSCs from different sources in a pre-clinical model relevant for the therapeutic application, by testing different batches of different MSC sources or by comparing pools of MSCs to avoid inter-donor variability. EV production is also influenced by the features of the producing cells. For instance, it has been shown that cell aging (replicative senescence and donor age-associated senescence) and cell–cell contacts (confluence and seeding density) affect EV production. Specifically, senescent MSCs secrete greater numbers of EVs than non-senescent MSCs (Huang et al., 2019; Fafian-Labora et al., 2020). Conversely, confluent MSCs produce lower amounts of EVs than proliferating MSCs (Patel et al., 2017). The impact of senescence on the production and functionality of MSC-EVs in different therapeutic applications has recently been reviewed (Boulestreau et al., 2020). EVs from aging MSCs did not exhibit the protective effect of EVs from young MSCs in an acute lung injury model (Huang et al., 2019). In consistency, intercellular transfer of EVs from young MSCs are more potent than EVs from aged MSCs to rejuvenate aged hematopoietic cells and restore their function through the uptake of autophagy-related mRNAs (Kulkarni et al., 2017). Production process will therefore have to include quality controls to evaluate the percentage of senescent cells and its impact on the functionality of EV batches.

Immortalized MSC lines could be used to ensure batch reproducibility, to avoid inter-individual donor variability, and to maintain bioactivity during culture expansion. For instance, EVs released by embryonic stem cell-derived MSCs immortalized by transfection of a lentivirus carrying the c-Myc oncogene reduced the infarct size in a mouse model of myocardial injury (Chen et al., 2011). Of course, the immortalized MSC stability and absence of the transgene protein in the derived EVs must be demonstrated. Nevertheless, this strategy ensures an infinite supply of EVs with high inter-batch reproducibility.

#### EV Production

A second question is the choice of culture system (e.g., medium composition and cell-adhering support) for EV production. Indeed, several cell culture parameters influence EV production and cargo composition. For clinical purposes, the use of xeno- and EV-free culture media is recommended to remove any source of variability and animal-associated contaminations. It has been shown that xeno- and serum-free culture media support sustained MSC proliferation without loss of viability and promote the cell secretory functions (Lee et al., 2017; Mochizuki and Nakahara, 2018; Palama et al., 2020). Platelet lysates can be used at the place of fetal calf serum in GMP manufacturing conditions, although defined media are more appropriate (Pachler et al., 2017; Bari et al., 2018). To scale up EV production for industrialization, 3D-culture in bioreactors has been tested, such as multilayered cell culture flasks, hollow fiber bioreactors, stirred-tank bioreactors, and spheroidal aggregates of MSCs. Hollow fiber and stirred-tank bioreactors are the more promising approaches because they are closed and GMP-compatible scalable systems that provide a high surface-to-volume ratio for MSC growth (Mendt et al., 2018; Mennan et al., 2019; Vymetalova et al., 2020). EV production in bioreactors is increased at least by 40-fold compared with 2D culture systems (Watson et al., 2016). Furthermore, the duration of EV production and the frequency of medium collection have to be tested to determine the optimal parameters for cell proliferation, confluence and EV re-uptake by producing cells.

EV production can be stimulated using different biochemical or biophysical strategies. Among the biophysical strategies, hypoxia can be controlled and modulated. It has been reported that MSC culture and EV production in hypoxic conditions (1–5% O_2_) increase the number of EVs released and their cargo composition (growth factors and miRNAs), thus enhancing their pro-angiogenic, immunomodulatory, cardioprotective and neuroprotective effects (Cui et al., 2018; Xue et al., 2018; Zhu et al., 2018). Another option is to take advantage of bioreactors to mechanically stimulate EV production by applying fluid shear stress or compression [for review, see Piffoux et al. (2019)]. Although, the underlying mechanisms are not known, one hypothesis is that in this condition, MSCs inhibit their own re-uptake of EVs. Recently, it has been reported that ultrasonication of ultracentrifuged MSC-EVs followed by regular centrifugation and filtration allows increasing the EV yield by 20-fold (Wang et al., 2019). Moreover, the authors demonstrated that these EVs are functional and promote wound healing in animal models. However, this technique might release a fraction of vesicles that normally remain tethered at the plasma membrane, or vesicles that have been recaptured by the producing cells, or even other components from secretory pathways (van Niel et al., 2018). Therefore, EVs isolated after ultrasonication need to be better characterized.

Different strategies have been considered to modulate EV content and biological activities, including biochemical stimuli and genetic modification of MSCs to overexpress specific proteins or miRNAs [for review, see Park et al. (2019)]. MSC activation with lipopolysaccharides before EV production does not change the number of released EVs, but influences their content. These EVs have been used to modify macrophage polarization, procoagulant properties, or the ability to support wound healing (Ti et al., 2015; Zeuner et al., 2016; Fiedler et al., 2018). The importance of miRNAs in MSC-EV therapeutic effects suggested that genetic engineering of MSCs to overexpress the miRNAs of interest might improve their efficacy. For example, miR-92a-3p overexpression in MSCs allowed producing EVs with higher protective effect against cartilage destruction in an osteoarthritis model (Mao et al., 2018).

In conclusion, all parameters that can influence EV number and content must be clearly identified to define the best balance between production conditions and EV functions. Improvement of EV functions can be obtained through genetic modification or pre-activation of MSCs. Because different manufacturing procedures and culture conditions can affect the characteristics and functionalities of EVs, the production process will have to be clearly defined for optimal use of EVs for specific clinical indications.

#### EV Isolation

There is no unique or standardized method to isolate EVs. This might explain the variability in EV characteristics and bioactivities among laboratories. For clinical applications, the challenge is to isolate EVs with high yield and purity, while preserving their structure and activity. In addition, the isolation method should be scalable, cost-effective, compatible with a high-throughput production process, and ideally, in a closed system. Differential ultracentrifugation-based techniques are the most common EV isolation methods in basic research, but they are not scalable, do not give pure EV preparations, may lead to EV aggregation, and are time-consuming. However, sequential centrifugation steps have been used for large-scale production of clinical-grade MSC-EVs (Mendt et al., 2018). Size-based fractionation methods that include tangential flow filtration and size exclusion chromatography are GMP-compliant and scalable systems for EV isolation [for reviews, see Agrahari et al. (2019); Paganini et al. (2019)]. Ultrafiltration also reduces the isolation times and costs compared with other techniques (Saxena et al., 2009; Bari et al., 2018, 2019b,c). In our opinion, currently, this is the method of choice for high-scale and GMP-compliant isolation of EVs. A comparative analysis of the secretome from BM- and AD-MSCs enriched by ultrafiltration or sequential ultracentrifugation indicated that ultrafiltration results in higher particle yield with higher protein content, in GMP-compliant conditions (Bari et al., 2019c). Finally, the choice of the isolation technique will have to be a compromise between EV yield and cost. In addition, it is important to keep in mind that each variation in the production process generates a product modification that will require a new functional qualification.

### Quality Controls

Quality controls concern MSC characterization and expansion, EV production and isolation, and the release criteria of EV batches [recently updated in Thery et al. (2018); Rohde et al. (2019)]. Attempts to standardize the methods of EV isolation and characterization are regularly discussed within ISEV. To measure the production yield, the number of isolated EV particles needs to be determined by Nanoparticle Tracking Analysis (NTA) or Tunable Resistive Pulse Sensing (TRPS). These methods allow measuring the number of particles in a solution. The production yield should be expressed as the particle number in cell equivalents because it takes into account the number of viable cells at harvest time and allows a better evaluation of the inter-batch reproducibility. Although there is no standard size for EV preparations, a size of ≤200 nm characterizes small EVs and could be defined as the standard to ensure better inter-batch reproducibility. EVs must also be profiled by flow cytometry or western blotting: expression of EV markers (CD9, CD63, TSG101, and CD81) and MSC markers (CD44, CD73, CD90, and CD105), and absence of signal for immune cell markers (CD14, CD34, and CD45). The presence of at least three different markers enriched in EVs should be a major criterion for batch release: CD9, CD63, CD81, Tsg101, Alix, and the ganglioside GM1, which has been described as an exosome marker (Tan et al., 2013). Finally, standard safety tests to exclude microbial impurities should be performed to determine the endotoxin levels, sterility, absence of mycoplasma, and absence of viral enrichment in the final product.

Additional information on protein and RNA concentration, which is not part of the released criteria, could be added to the quality control list. This information allows expressing the number of EVs as particles per μg of protein or RNA, and could be used to assess inter-batch reproducibility. In addition, specific microRNAs or proteins, known to be relevant for EV therapeutic effect, could be identified and quantified by quantitative PCR and ELISA assays to provide supportive data on EV functional properties. This will be relevant to define potency assays for EVs in relation to the dedicated clinical applications.

### Storage and Stability

The preservation of EV biological activity during storage is both critical and challenging. Few studies have reported consistent data on EV storage and formulation. Siliconized vessels are recommended for EV storage to prevent their adherence to surfaces and their loss (Jeyaram and Jay, 2017). Phosphate buffered saline is habitually used for EV resuspension. Storage at −80°C is encouraged, although it can affect EV size, number and function (Lorincz et al., 2014; Cosenza et al., 2018). It has been reported that EV concentration (quantified by NTA) remains stable after 1 week of storage at +4, −20, and −80°C (Jeyaram and Jay, 2017). Nevertheless, storage at +4°C causes EV aggregation, and the amount of the associated proteins and miRNAs dramatically decreases at +4°C and −20°C. For clinical applications, EV products need to be suspended in sterile 0.9% NaCl and stored at −80°C. Moreover, they should be frozen and thawed rapidly to preserve their morphology and function. EV products should be formulated for single-use because it has been observed that their number decreases, and their morphology and content are altered after two cycles of freezing and thawing (Kusuma et al., 2018).

The possibility to freeze-dry the EV products for long-term storage at room temperature has been investigated. Freeze-drying preserves EV characteristics and function, and thus might represent a cost-effective storage strategy. It also reduce transport costs (Charoenviriyakul et al., 2018). The characteristics and functionality of peripheral blood mononuclear cell-derived secretomes remain stable for up to 6 months after lyophilization and high dose γ-irradiation when stored between −20 and +25°C (Laggner et al., 2020). However, such lyophilized secretomes contained albumin, cholesterol and triglycerides that might have preserved the sample bioactivity. Another study showed that the exosome number and size distribution and biological activity are not affected after storage at −80°C for 45 days or 6 months (Mendt et al., 2018). Disaccharide stabilizers could be added in the storage buffer to improve EV preservation. Trehalose is a natural, non-reducing disaccharide sugar used as a cryo-preservative for labile protein drugs, vaccines, and liposomes. Its safety and tolerance have been demonstrated in mice and humans after oral, gastric and parenteral administration (Sato et al., 1999; Richards et al., 2002). It has been reported that addition of trehalose to EV samples improves their stability when stored at −80°C and when lyophilized, by preventing EV aggregation and lysis (Bosch et al., 2016; Charoenviriyakul et al., 2018). Mannitol is another cryoprotectant that maintains the functionality of freeze-dried secretomes stored at −20°C for at least 2 months (Bari et al., 2019c). The addition of 5–10% dimethylsulfoxide (DMSO) also maintains EV integrity and function (Romanov et al., 2019). The possibility to develop an off-the-shelf lyophilized product is a huge strength compared with the parental cell product that must be frozen for preservation and must be transported fresh after revitalization and/or expansion, or frozen under stringent requirements. Finally, whatever the storage formulation and conditions, batch stability will have to be carefully examined and monitored during storage. Stability can easily be assessed by quantifying the particle number, the quantity of total RNA and proteins, and the MoA-associated bioactive factor at different times during storage.

## Regulatory Aspects for the Industrial and Clinical Use of Extracellular Vesicles

The regulatory aspects for manufacturing and clinical applications of EVs as new therapeutics have to be implemented. In 2015, an ISEV position paper discussed the classification of EV-based products as biological medicine or biological drugs, and categorized EVs based on the anticipated active substance(s) (Lener et al., 2015). It also provided a detailed discussion on the regulatory issues associated with EV-based therapeutics. According to the regulatory frameworks for manufacturing and clinical trials in Europe, United States and Australia, quality and safety control data must be provided, as underlined in the previous section. In addition, the existing guidelines require the identification, quantification and characterization of the main substance(s) of a biological drug to indicate the MoA. The active substance determines the pharmaceutical classification and the MoA, and will define the potency assay to be used (Rohde et al., 2019). However, we can expect that the MoA will not be limited to a single molecule, as it has been shown for MSCs, and it will be difficult to precisely define the active substance in EVs. We can also anticipate that the MoA, and associated bioactive factors, of a same MSC-EV batch may also depend on the targeted clinical application and the related therapeutic function. Interestingly, a review paper discussed the respective role of miRNAs and proteins as major factors in the MoA and in mediating EV therapeutic effect (Toh et al., 2018). A prerequisite for their potency is the presence of biologically relevant amounts of molecules. By analyzing the average quantity of miRNAs in MSC-EVs and the possible number of EVs taken up by a cell, the authors concluded that miRNAs are not likely to be in the right concentration or configuration to have a relevant biological activity. A similar analysis for proteins indicated that they were more likely to elicit a biologically relevant response, suggesting that proteins could be the main drivers of MSC-EV MoA. Nevertheless, it has been reported that several miRNAs are important actors, at least as mediators, of MSC-EV immunoregulatory effects (Martin-Rufino et al., 2019). More studies are needed to bring firm conclusions on the role of proteins and miRNAs in the MoA of MSC-EVs. Although not required in the early stages of clinical development, the definition of the active substance(s) that supports the MoA and the efficacy of EV-based treatments is a requirement to develop appropriate pharmaceutical control strategies. Indeed, in the early phases 1 and 2 of pharmaceutical development, the batch-to-batch consistency must be checked using biochemical, biophysical and functional assays (Rohde et al., 2019). Due to their complex nature, the specific MoA of EVs may be difficult to identify; however, in contrast to cells, it could be easier to set-up quality control tests for EV characterization and inter-batch homogeneity assays (Riazifar et al., 2017). The regulatory requirements will also be different for EV-based drugs derived from cells (genetically engineered or not) and for EVs used as drug-delivery systems [for review see Lener et al. (2015)]. Compliance with the regulatory frameworks is pivotal for the approval of EV-based therapies and their large-scale implementation.

## Conclusion: Challenges and Perspectives

MSC-EVs exert comparable therapeutic functions as their parental cells, but have some advantages over MSCs because they lack nuclei and cannot abnormally proliferate or differentiate. Moreover, small-size EV preparations that are isolated using protocols including a filtration step through 0.22 μm membranes can be considered as sterile and do not require an additional sterilization step. However, there are still many challenges to be addressed concerning the scalable production, standardization, and characterization of EV products for the successful translation of EV-based therapeutics in the clinic (Agrahari et al., 2019). The heterogeneity of MSCs used for EV production (BM, adipose tissue, other tissues; non-manipulated or immortalized) and of the obtained EVs (production process; EV size; contents of EV fractions) makes difficult to select the EV drug with the highest therapeutic efficacy. Several companies have already developed EV- or secretome-based products for different clinical applications using diverse cell sources, and MSC-derived EVs represent around 40% of such products (Gimona et al., 2017). The best sources for reproducible, safe and cost-effective production must be identified using the relevant non-clinical models for each specific clinical applications. Large-scale processes to manufacture EV therapeutics in GMP conditions (mainly bioreactor technologies for EV production, and ultrafiltration technologies for EV purification) are being implemented using preferentially closed systems for higher safety to ensure robust production procedures. Several GMP-compliant processes for the production of MSC-EV or secretome products have been developed (Pachler et al., 2017; Bari et al., 2018; Mendt et al., 2018; Laggner et al., 2020). Importantly, although quality controls for cell production include cell viability and apoptosis rate measurements, they do not assess cell senescence. Yet, EV yield is higher when using senescent cells, and their cargo composition is altered (abnormal levels of some miRNAs) [for review, see Boulestreau et al. (2020)]. The proportion of senescent cells in the production batches and their effect on EV content should be taken into account in quality control procedures. Moreover, EV standardization (protein or RNA quantification, particle determination) should be improved. In addition, some undesirable miRNAs, such as miR-410 that promotes carcinoma cell growth and aging-associated miRNAs, should be quantified (Fafian-Labora et al., 2017; Dong et al., 2018). Analytical methods to accurately characterize EVs at the single-vesicle level are under development and are needed for reliable standardization. Lyophilization could be used for the long-term storage of EVs and to develop off-the-shelf products with high stability. This would represent a real advantage compared with MSCs because it would facilitate and reduce the costs of storage and transport (at room temperature). The formulation of EVs into a standardized biological drug has to be defined for each clinical application in terms of dosage, excipients (use and type of cryoprotectant, for example) and pharmaceutical forms (powder or liquid) (Bari et al., 2019b). The formulation may depend on the administration route. While liquid formulations can be used for systemic or parenteral injection of EVs, powder formulations might be preferred for oral or aerosolized administrations. The dosages for a specific application will determine the batch sizes for production (Rohde et al., 2019). The procedure for the characterization of the active substance(s), which can be localized in the inner and/or outer part of EVs, and of the “excipient” non-biologically active moiety of the EVs will have to be established before the industrialization step. It is now crucial to address the challenges related to the production and the regulatory and clinical aspects of EV-based biological products in order to pave the way to their commercialization.

## Author Contributions

All authors contributed to the design and writing of the manuscript. MM, PR, JB, and CJ proofread and given comments as well as suggestions. DN supervised and finalized the manuscript.

## Conflict of Interest

MM is an employee of Bauerfeind France. The remaining authors declare that the research was conducted in the absence of any commercial or financial relationships that could be construed as a potential conflict of interest.
